# Electrical and Thermal Conductivity of Epoxy-Carbon Filler Composites Processed by Calendaring

**DOI:** 10.3390/ma12091522

**Published:** 2019-05-09

**Authors:** Andrea Caradonna, Claudio Badini, Elisa Padovano, Mario Pietroluongo

**Affiliations:** Department of Applied Science and Technology, Politecnico di Torino, 10129 Torino, Italy; andrea.caradonna@polito.it (A.C.); elisa.padovano@polito.it (E.P.); mario.pietroluongo@polito.it (M.P.)

**Keywords:** calendaring, electrical conductivity, thermal conductivity, hybrid composites

## Abstract

Electrical and thermal conductivity of composites which contain carbon-based fillers in an epoxy matrix were investigated. The fillers were dispersed in the liquid matrix by using three roll mill equipment. The filler/matrix mixture was cast in a mold and then cured, thus obtaining composite specimens. Multiwall carbon nanotubes, graphene-like nanoplatelets, and graphite were used as fillers and their effect on conductivity was investigated. Electrical and thermal conductivity were measured at different filler loads. It was found that the formation of percolation paths greatly enhanced electrical conductivity, although they were not so effective in improving thermal conductivity. The behavior of composites containing each single filler was compared with that of hybrid composites containing combinations of two different fillers. Results show that fillers with different aspect ratios displayed a synergetic effect resulting in a noticeable improvement of electrical conductivity. However, only a small effect on thermal conductivity was observed.

## 1. Introduction

Carbonaceous materials like carbon black and graphite are traditionally used as filler for polymers because they improve some mechanical properties and reduce the cost of the materials. Over the last twenty years nanostructured carbonaceous fillers, in particular carbon nanotubes (CNTs) and graphene or graphene-like nanoplatelets (GNPs), have attracted increasing interest as components of polymer-based nanocomposites. The remarkable mechanical, electrical, and thermal properties of these kinds of nanofillers offer enormous potential for the production of composites showing high performance [1,2,3].

Although Thostenson et al. [4] claimed that multiwall carbon nanotubes (MWCNTs) have a theoretical elastic modulus exceeding 1 TPa, direct tests with an atomic force microscope measured an elastic modulus ranging from 270 to 950 GPa, and tensile strength ranging from 11 to 63 GPa [5,6,7]. Measurements taken on a graphene single layer using a diamond based atomic force microscope tip gave results of an elastic modulus of 1TPa and a tensile strength of 130 GPa. These values, which are in good agreement with theoretical computer simulation [8,9], are due to the strong covalent C–C bonds inside graphene layers, and therefore are characteristic of a single graphene platelet and a single nanotube. Unfortunately, both the elastic modulus and tensile strength diminish when the number of layers of GNPs increases, thus progressively approaching the mechanical features typical of graphite. 

Also, electrical and thermal conductivity of single carbon nanofillers are very good. In fact the electrical conductivity of a single MWCNT ranges between 10^5^ S/m and 10^7^ S/m, while 10^5^ S/m is a typical value of conductivity for a GNP [3,10,11]. Thermal conductivity values of 2000 W/(m·K) and 5000 W/(m·K) are generally accepted for a MWCNT and GNP, respectively [10,11]. Thermal conductivity of these nanofillers is much better than that of graphite (298 W/(m·K)) which, on the contrary, shows similar electrical conductivity. Additionally, very different values of thermal conductivity can also be found in the literature for MWCNTs and GNPs since their conductivity is strongly affected by the presence of defects, the production process, the number of planes in the GNPs, and the measurement method [3,12,13,14]. For instance, it was found that the defects induced by electron beam irradiation reduce the thermal conductivity of graphene by 70% [15]. In addition, both electrical and thermal conductivity of graphene-like nanoplatelets are very high in the plane, but they dramatically decrease in the direction perpendicular to the graphene planes, as happens for graphite too [11]. Finally, when a network of CNTs or GNPs is considered, the resistance of the interfaces greatly lowers the conductivity.

The homogeneous distribution of the nanofillers within the matrix is required for exploiting at best their potential, and therefore a great effort has been made to improve the production process of nanocomposites. Unfortunately, these fillers are prone to form particle aggregations or bundles of nanotubes owing to the very high Van der Waals attractive forces. For this reason, the main problem to face when processing nanocomposites deals with the separation of nanoparticles/nanotubes from their aggregates. Several processing methods have been investigated for polymer nanocomposites fabrication. For instance, the most suitable methods for thermoplastic matrix composites consist in the production of a mixture of filler and matrix followed by injection molding. Melt-mixing or solution compounding (which consists in the dissolution of the polymer in a solvent and the subsequent dispersion of the filler in the liquid with the help of stirring or sonication) can be adopted for the production of the filler/matrix mixture [3,16]. Several methods have been also developed for the dispersion of nanofillers in thermoset polymers, which are liquid before curing. The nanofiller can be added to the liquid resin and dispersed by stirring, high speed mixing, sonication, calendaring, or a combination of two of these techniques [3,17,18,19,20,21]. Frequently a solvent is also added to the resin in order to decrease the viscosity and make the dispersion easier. The use of solvents for both thermoplastic and thermosets matrices favors the dispersion process, but solvents must be evaporated at the end of the process (after casting), and re-aggregation of the nanofiller can occur during evaporation.

Mixing performed using high-shear mixing or sonication frequently causes the breakage of the fillers and the resulting reduction of the length of CNTs or the lateral size of GNPs [22].

Calendaring, performed by using three roll mill equipment, is a well-established and gentle process based on the adoption of shear stresses. According to this process, after the dispersion of nanofillers in liquid matrices (such as epoxy or silicone), the addition of the curing agent, and the casting in a mold, the material is submitted to a final curing treatment [20,21,22,23]. The possible damage of the nanofiller during the mixing step can be avoided by selecting proper processing parameters that, at the same time, allow a homogenous distribution of the filler inside the resin. The carbon nanotubes tend to align parallel to the rolling direction. Similar effect of preferred orientation can be observed for graphene-like platelets too, but not for every GNP load [21]. Additionally, an anisotropic conductivity of the material is expected to result from the texture caused by calendaring [21]. In addition GNPs can suffer exfoliation during the calendaring process [21]. The nanofiller/liquid matrix blend shows viscoelastic behavior: Dispersion occurs owing to suitable shear stresses, but when the shear stress decreases below a threshold the viscosity greatly increases, which hinders the re-aggregation of the nanotubes or the platelets [20]. 

It is generally accepted that carbonaceous fillers grant good conductivity when their concentration inside the polymer matrix exceeds a critical concentration (called percolation threshold). The achievement of the percolation threshold for electrical conductivity surely occurs when the nanotubes or the conductive nanofillers physically touch each other, but conductivity was also observed when the distance between the nanotubes decreases to about 5 nm, because a tunneling mechanism operates [20].

When the fillers align in one direction a continuous network of CNTs or GNPs more easily forms. This effect can be achieved by applying shear forces, as well as by using electrical or magnetic fields [24,25,26].

In the case of GNPs, the size of the platelets can also greatly affect electrical and, mostly, thermal conductivity. The composite conductivity increases when the platelet size increases [21]. In fact, the interfacial thermal resistance plays a dominant role in the thermal conductivity and, for this reason, it is convenient to use large graphene-like sheets in order to limit the effect of the high thermal boundary resistance.

According to recent research advances [2,17,27], the addition of carbon nanoparticles (such as graphene platelets or carbon black) to CNT-polymer composites can provide a synergetic effect, which results in further improvement of mechanical features [28,29], thermal conductivity [30,31], electrical conductivity [32,33], and piezoresistive behavior [34]. On the contrary, other authors did not observe any synergetic effect in these kinds of hybrid composites [35]. The synergetic effect on the electrical conductivity is believed to arise from the combination of two conducting fillers with different geometrical shapes and aspect ratios, which results in the formation of continuous GNP-CNT-GNP or CNT-GNP-CNT network structures in the polymer [33,36]. Graphene acts as a “spacer” to separate entangled CNTs, and CNTs bridge the gap between graphene sheets or carbon nanoparticles. In addition, these network structures can give rise to strong π–π interaction with benzene rings and oxygen atoms possibly present in the polymeric chains constituting the matrix [36]. It was proposed that both mechanical and electrical properties appreciably depend on the ratio between the concentrations of the two types of nanofillers, but this ratio does not affect all these properties in the same manner. For this reason no best ratio exists which can optimize both mechanical and electrical behavior [34,37], and the hybrid nanocomposites should be designed for each specific application. 

The above-mentioned hybrid composites offer the chance of greatly decreasing the total content of carbonaceous fillers in conductive polymers, and reducing the material cost by replacing the rather expensive nanotubes with cheaper fillers. Actually, ultrahigh volume fractions (40–50 vol.%) of CNTs or GNPs or their combinations can result in a significant increase of thermal and electrical conductivity [17], but the dispersion of such a high quantity of filler is very difficult. With the increase of the filler content, agglomeration phenomena and presence of defects (such as porosity) hardly can be avoided. This kind of defects in the microstructure greatly worsens the mechanical behavior. 

The present work was aimed at producing composites with very small amount of fillers and good electrical and thermal conductivity, obtained by exploiting the filler synergistic effect and an optimized processing path. Combinations of CNTs with graphite and graphene were investigated. An epoxy resin was used as a matrix since this kind of material allows to exploit all the advantages that the calendaring process offers. 

## 2. Materials and Methods

The nanocomposites were processed by using an epoxy resin as a matrix (Technovit®, Kulzer, Wehrheim, Germany), an amine curing agent (resin: hardener ratio = 2:1, polymer density of 1.15 g/cm^3^ after curing) and different fillers, showing the following characteristics according to the technical data sheets of the manufacturers:

- Multi-walls carbon nanotubes NC7000^TM^ (produced by Nanocyl SA (Sambreville, Belgium) through chemical vapor deposition process) with an average length of 1.5 µm, diameter of 9.5 nm, electrical conductivity of 10^6^ S/m, and density of 1.72 g/cm^3^.
- Graphene nanoplatelets GAbcr (purchased from ABCR Gute Chemie, Karlsruhe, Germany) with size of 15 μm, 6–8 nm thick, and density of 2 g/cm^3^.
- Natural graphite flakes, (purchased from Alfa Aesar, Haverhill, MA, USA) with median size from 7 to 10 µm, purity level of 99% (metal basis), and density of 2.2 g/cm^3^ [11].

Samples of 45 g each were obtained by mixing the resin with the curing agent and the fillers by means of a three roll mill apparatus, casting the mixture in a silicon cylindrical mold and curing for 12 h at room temperature. The three roll mill consists of three rolls rotating in opposite ways; the mixture is fed between the first and the second roll while a scraper allows to collect the final mixture from the third roll. This apparatus can operate according to different modes: gap mode and contact mode. In gap mode, it is possible to modulate the distance between the rolls in order to vary the shear forces. This distance can be varied considering that the gap between the first and the second roll, (gap 1), which must be three times greater than the gap between the second and the third roll (gap 2). The minimum gap between a couple of rolls is 5 µm (when operating in the gap mode).

In the contact mode, the rolls are in contact with each other, thus providing the highest shear stress to the system. Also, the rotation speed of the rolls affects the resulting shear stresses. The speed of the rolls is linked by 1:3:9 ratios in order to grant a continuous flow of material through the apparatus. The third roll speed can be changed in a continuous manner, but the maximum rotation speed is 600 rpm in the gap mode and 300 rpm in the contact mode. 

The dispersion of filler within the epoxy matrix by the three rolls mill was formerly investigated by processing epoxy/GNP samples with 2 wt.% of filler and using different parameters. The resin and the fillers were first mixed by mechanical stirring and then processed by a mini-calendaring system (Exakt 80E). The quality of the resulting samples was compared through the measurement of their thermal conductivity. In this manner, the best processing parameters were selected and adopted for the preparation of all the composites under investigation, as described in the following. The speed of the third roll was fixed at 600 rpm. The mixing process consisted of six steps carried out with different gap sizes between the rolls. The initial three steps were performed using a gap of 45 μm between the first and the second roll, and of 15 μm between the second and the third roll. The further three steps were performed using gaps of 15 μm and 5 μm between the couples of rolls. 

Binary composites (consisting in matrix and a single filler) with different filler concentrations were processed: from 0 to 1 wt.% of CNTs, from 0 to 30 wt.% of GNPs, and from 0 to 45 wt.% of graphite. Four sets of ternary hybrid composites (CNTs plus GNPs, or CNTs plus graphite added to the matrix) were also prepared. These hybrid composites contained 0.1 wt.% or 0.05 wt.% of CNTs, coupled with a concentration of GNPs or graphite progressively increasing from 0 to 5 wt.%.

Bars 20 × 10 × 2 mm^3^ in size and discs with a diameter of 20 mm and a thickness of 10 mm were obtained from the cylindrical composite samples by cutting them with a diamond blade perpendicularly with respect to their height. These samples were used for electrical and thermal conductivity measurements, respectively. For each kind of composite (that is for each composition investigated) the electrical conductivity and the thermal conductivity were measured on two or five samples, respectively. The extremities of the bars were painted with a silver-based conductive paint and the electrical resistance was measured by the 2-point method using an Agilent 34420A NanoVolt/Micro-Ohm Meter equipment (Agilent Technologies, Santa Clara, CA, USA). The measured data were normalized on the bar size (length and thickness) obtaining the values of resistivity. Few samples for electrical conductivity measurements (bars with the same size of specimens described above) were also taken in the direction parallel to the cylinder axis. The thermal conductivity was investigated at room temperature using a Hot Disk Thermal Constants Analyzer equipment (Hot disk TPS 2500, Hot Disk, Göteborg, Sweden).

The density of composites was measured by Archimede’s method. The cryofractured surfaces of the composites were observed by using field emission scanning electron microscopy (FE-SEM, Zeiss Merlin equipment, Oberkochen, Germany). To avoid charging, a few nanometers-thick layer of chromium was deposited on samples. 

## 3. Results and Discussion

### 3.1. Optimization of Three Roll Mill Process

Several mixing trials were carried out with the aim of optimizing the processing parameters and obtaining a homogeneous dispersion of the filler within the matrix by using the minimum number of steps. The homogeneous dispersion of the filler is expected to promote the formation of a conductive network and then to enhance both thermal and electrical conductivity. In addition, the homogeneous dispersion of filler would avoid agglomeration of filler particles, which is detrimental for mechanical properties. The value of the thermal conductivity was taken as an indicator of the effectiveness of the dispersion process. Each trial consisted of several steps (repeated treatments of the mixture by using the apparatus) performed using different roll speeds and gaps. The severity of the processing method was progressively enhanced by increasing the number of steps, decreasing the roll gap, and adding passages in contact mode. The conditions adopted for these processing trials and the resulting thermal conductivity of the composites with 2 wt.% GNPs are shown in Table 1.

Table 1 shows that the best thermal conductivity was achieved using the processing conditions of trial “B”.

The addition of further steps in gap mode (trial C) or in both gap and contact modes (trials D, E, F) did not result in any improvement of thermal conductivity, which decreased with respect to trial B. The microscopic examination of the samples showed that a rather good distribution of filler inside the matrix can be achieved adopting the conditions of trial “B”, while the increase of the number of steps, in particular of those performed in contact mode, can cause the reduction of the platelet size. Very likely the damage of the filler overbalances the improvement of homogeneity of filler distribution. As discussed in the next paragraph, the calendaring process can also result in the preferred orientation of some fillers, which can make the conductivity depending on the direction. 

### 3.2. Composite Microstructure-Filler Orientation

**Figure 1:**
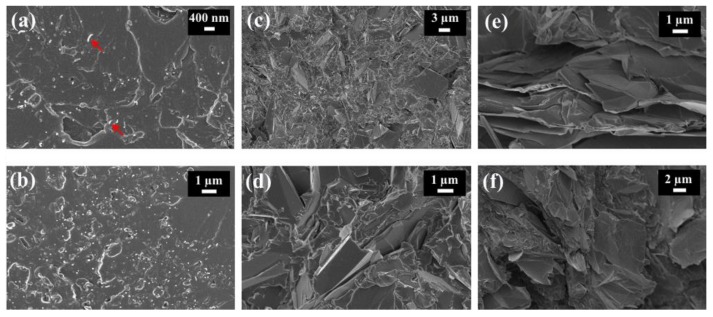
Fracture surface of composite with 0.2 wt.% of carbon nanotubes (CNTs) 50k× (**a**), 1 wt.% of CNT 25k× (**b**), 40 wt.% of graphite 5k× (**c**), 40 wt.% of graphite 25k× (**d**), 6 wt.% of GNP 25k× (**e**), 25 wt.% GNP 10k× (**f**).

The fracture surfaces of composite samples, obtained from the bars used for electrical resistance measurements and then machined in the direction perpendicular to the cylindrical cast samples, are depicted in Figure 1. All these fracture surfaces represent a cross section of the bars.

The fracture surfaces of the composites containing 0.2 wt.% and 1 wt.% of CNT (Figure 1a,b) show several tubes perpendicular to the surface and rather well distributed within the matrix. Some of them (put in evidence by red arrows) are protruding out of the surface and suffered pull-out before the sample fracture. These pictures prove that calendaring causes a certain degree of CNT alignment. In contrast, the three roll mill process did not result in the preferred orientation of graphite flakes, since they seem randomly oriented on the fracture surface (Figure 1c,d). A certain effect of orientation was observed, instead, for the composites containing rather low percentages of GNP (Figure 1e), while this texture became less marked when the content of GNP increased (Figure 1f).

**Figure 2:**
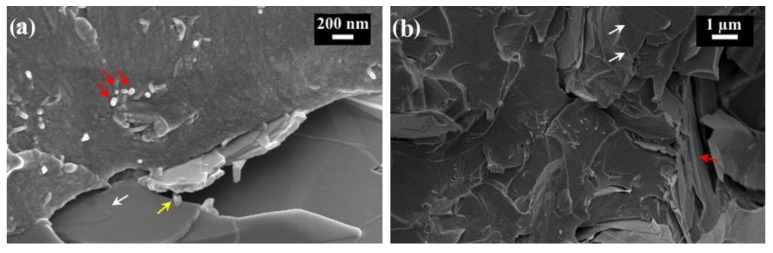
Fracture surface of composite with 0.1 wt.% of CNT plus 5 wt.% of graphite (**a**) and 0.1 wt.% of CNT plus 5 wt.% of GNP (**b**).

The different effect of calendaring on preferential orientation of fillers with different aspect ratios and size can be also appreciated on the fracture surface of the hybrid composites, for example, on that of the composite containing 0.1 wt.% of CNT and 5 wt.% of graphite (Figure 2a). In this picture CNT are placed roughly perpendicularly to the surface (red arrows) and graphite plates lying on the surface (white arrows) can be seen. Nanotubes bridging graphite plates can be also observed (yellow arrows).

Also in the case of hybrid CNT + GNP composites (Figure 2b) the alignment of CNT is well evident, while only some GNP are placed perpendicularly to the fracture surface (red arrow) and others are parallel to it (white arrows).

Some porosity was observed by microscopy in the composite samples. The densities of some representative samples are summarized in Table 2 and compared with the theoretical ones, calculated according to the rule of mixture. From these results, it is evident that the measured density was generally lower than that expected based on the sample composition and the density of filler and matrix. This deviation from the theoretical density progressively increased with the amount of filler load, in particular when graphite was used as a filler. It is very likely that the increase of filler concentration made the filler dispersion more difficult and favored the agglomeration of the filler particles, which may have resulted in the increase of the porosity level and in the decrease of both electrical and thermal conductivity.

### 3.3. Electrical Conductivity

**Figure 3:**
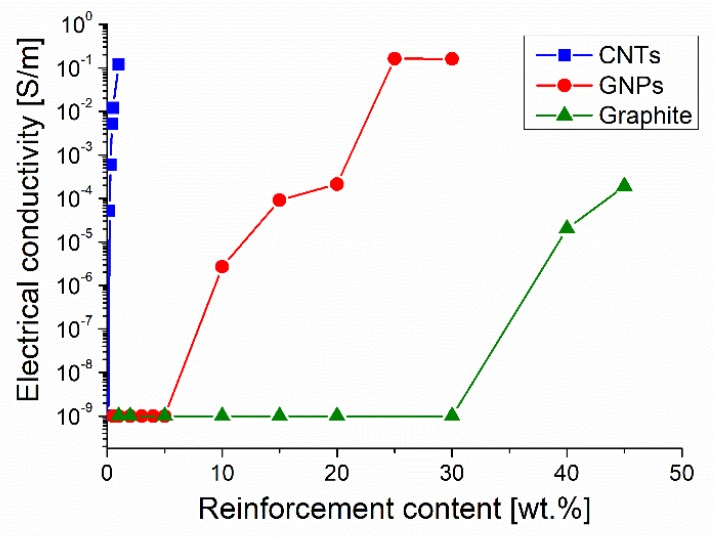
Effect of the filler content on the electrical conductivity of composites.

The electrical conductivity of two-components and three-components materials is shown in Table 3. Figure 3 shows that conductivity changes with the concentration of CNTs, GNPs, and graphite; very different effectiveness can be observed for these three fillers.

The best conductivity was achieved using a very low concentration of CNTs (1 wt.%), while a similar electrical conductivity value was obtained by using GNPs as filler but in a much higher concentration (25 and 30 wt.%). The addition of graphite also resulted in a more limited conductivity increase when a huge load of this filler was adopted. These results are consistent with the higher intrinsic conductivity of MWCNTs (10^6^ S/m) with respect to that of either GNP (10^5^ S/m [21,23]) or graphite (10^5^ S/m [22]). In Figure 3 percolation thresholds at 1.34 × 10^−1^ vol.% (0.2 wt.%), 9.21 vol.% (15 wt.%), and 21.96 vol.% (35 wt.%) can be observed for MWCNT, GNP, and graphite respectively. 

The alignment of MWCNTs in the direction along with the conductivity measured very likely concurs to enhance their capability to increase electrical conductivity. It is generally accepted that when a conductive filler is dispersed in an insulating polymer, electrical conductivity can be achieved only when the filler concentration is high enough to form a conductive network of physically interconnected carbonaceous elements (plates or tubes). When these chains of conductive elements are placed in a plane, or along a direction, the effect of the conductive elements is maximized in the plane or direction of alignment, but the material shows strong anisotropy. On the other end, only a fraction of the conductive elements probably takes part in the formation of the conductive paths, and very likely not all the conductive chains extend over the full length of the composite samples. For these reasons, it is quite hard to find a simple correlation between the filler concentration and the real electrical conductivity. In addition, the electrical conductivity of chains of conducting carbon tubes or plates is expected to be lower than that of the single tube/plate, which can be calculated or measured. These characteristics of nanocomposites make it difficult to predict their electrical behavior. Several predicting models have been proposed, but to our knowledge, there is not yet a method of general use. Models based on conductivity and the nominal volume fraction of the filler as input data generally bring to conductivity values well over the experimental ones, unless semi-empirical parameters are introduced in the calculation to fit the experimental behavior [38].

The preferred orientation of CNTs in our samples processed by three roll mill was confirmed by comparing the conductivity values measured in the directions parallel and perpendicular with respect to that of MWCNT alignment. In samples containing 1 wt.% of MWCNTs, the electrical conductivity measured in the direction of tube alignment was 1.2 × 10^−1^ S/m, while it decreased down to 5 × 10^−5^ S/m in the perpendicular direction (parallel to the height of cylindrical cast samples).

The significant difference in electrical conductivity observed between the composites containing GNPs and graphite can be explained with the tendency of GNPs to align in the rolling direction and lie on the rolling plane while the graphite plates show a random orientation. In fact, the conductivity of both GNPs and graphite is much higher in the graphitic planes than in the thickness direction. Therefore, it is necessary to greatly increase the content of graphite to obtain a conductive material; on the other hand, the composite processing becomes more and more difficult with the increase of filler concentration and this results in an increased amount of porosity, which limits the effect of the high filler concentration.

The electrical conductivity of 1.2 × 10^−1^ S/m measured for composites containing 1 wt.% (0.67 vol.%) of MWCNTs is consistent with the literature data reporting the conductivity of epoxy/MWCNT composites processed by three roll mill (10^−2^ S/m using 1.35 wt.% of MWCNTs [20]) or by shear mixing (10^−1^ S/m using 1.0 wt.% of MWCNTs [19]). On the contrast, the electrical conductivity obtained by using three roll mill process was better than that reported in the literature for similar composites with much higher MWCNT content, but processed by sonication or simultaneous magnetic stirring and sonication: 2 × 10^−2^ S/m when using 10 vol.% of filler [17] and 1 × 10^−3^ S/m when using 2 wt.% of filler [18], respectively. Then the processing method seems to entail great importance for the conductivity of epoxy/MWCNT composites. 

Also, literature data about epoxy-GNP nanocomposites strengthen the importance of the processing method on the electrical conductivity. For instance, Chandrasekaran et al. [39] obtained a conductivity of 1.8 × 10^−3^ S/m for an epoxy/GNP composite with 1 wt.% of GNPs 20–50 µm in size processed by three roll mill, and claimed that this conductivity was three orders of magnitude higher than that obtained for the composite with the same concentration and kind of GNPs, but produced by sonication combined with shear mixing. Huang et al. [17] produced by sonication epoxy/GNP composites and had to increase the concentration of GNPs (5 µm) up to 15 wt.% to obtain a similar conductivity value of about 2 × 10^−3^ S/m. In the present investigation, when using three roll mill and 25 wt.% of GNPs, 15 µm in size, a conductivity of 1.6 × 10^−1^ S/m was achieved, which is better than the conductivity (about 10^−2^ S/m) observed by Huang et al. for a composite processed by sonication with the same concentration of GNPs, 5 µm in size. On the other hand, in the present investigation the percolation threshold was observed at a GNP concentration well over that reported by Chandrasekaran et al. [39], which can be attributed to the different kind of GNPs used (size and possible presence of defects in this filler).

**Figure 4:**
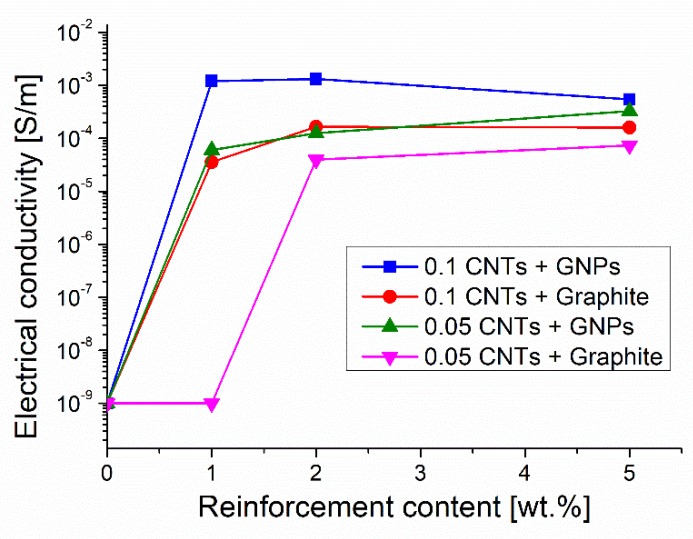
Synergetic effect of filler combination on the electrical conductivity of hybrid composites.

The combination of fillers with different aspect ratios (MWCNT + GNP and MWCNT + graphite) resulted in a synergetic effect, as depicted in Figure 4.

Figure 4 shows that by simply adding 1 wt.% of GNPs to a composite containing 0.1 wt.% of CNTs it is possible to exceed the percolation threshold and obtain appreciable electrical conductivity values (1.2 × 10^−3^ S/m.) Nevertheless, graphite was less effective than GNPs because the addition of 1 wt.% of this filler to the epoxy-0.1 wt.% of the CNT system resulted in an electrical conductivity of 3.57 × 10^−5^ S/m only; a further conductivity improvement was achieved when adding 2 wt.% of graphite. In every case the percolation threshold for hybrid composites was reached when the content of CNTs was a half of that required for the percolation threshold observed when CNTs are alone, moreover it was reached when the content of GNPs or of graphite was about 2%–3% of the amounts these fillers needed to get to the percolation threshold when they are alone. The synergetic effect can be also observed when GNPs or graphite are added to an epoxy/CNT composite containing 0.05 wt.% of CNT. In this last case, good conductivity was achieved when 2 wt.% of GNPs or graphite was added. On the other hand, further additions of GNPs or graphite (over 1 wt.% or 2 wt.%, respectively) to the composites containing 0.1 wt.% or 0.05 wt.% of CNTs did not result in an appreciable increase of conductivity. In fact, for instance, not important electrical conductivity gain was observed when the GNPs or graphite content was enhanced up to 5 wt.%. This result seems to enlighten the main role of CNTs, and it suggests that synergistic effect can be achieved as soon as a proper ratio between the concentration of CNTs and other fillers is accomplished. In addition, the more convenient proportion between CNTs and the second filler was found to depend on the actual CNT concentration. A 1:10 weight ratio between CNTs and GNPs or graphite resulted in the best conductivity when the CNT content was 0.1 wt.%, while the best ratio increased up to 1:40 when the content of CNTs was reduced to 0.05 wt.%.

The adoption of three rolls mill for the filler dispersion seems more convenient than sonication or contemporaneous stirring and sonication also when hybrid epoxy-MWCNT-GNP composites are processed. The hybrid composite with 0.1 wt.% CNTs (0.067 vol.%) plus 1 wt.% GNPs (0.58 vol.%) showed a superior electrical conductivity (1.2 × 10^−3^ S/m) than a similar material processed by stirring and sonication (10^−5^ S/m) and containing 1.6 wt.% CNTs plus 0.4 wt.% GNPs [18]. The electrical conductivity achieved in the present investigation for this composite was not too far from that observed for a sonicated composite with much higher filler content (2 × 10^−2^ S/m, obtained by using 5 vol.% CNTs plus 5 vol.% GNPs) [17]. Also in the case of hybrid composites with CNTs plus graphite produced by three roll mill, the results were consistent with those reported for similar shear stirred materials [19].

A general equation for the prediction of the formation of a conductive network in hybrid composite systems (containing CNTs and another carbon-based filler) has been proposed and widely used to explain their electrical behavior [2]:

(1) $$\frac{\;V_{CNTs}}{\varphi_{CNts}}+\frac{V_{cf}}{\varphi_{cf}}=1$$

In Equation (1) $V_{CNTs}$ and $\varphi_{CNts}$ are respectively the actual volume fraction of CNTs in the hybrid composite and the volume fraction necessary for achieving the percolation threshold when CNTs are alone (not hybrid composites). Similarly, $V_{cf}$ and $\varphi_{cf}$ have the same meaning, but they are referred to as the second carbonaceous filler. It has been proposed that when the two conductive fillers combine with each other to form a conductive network the value of the equation becomes equal or higher than one as soon as the percolation threshold is reached or exceeded. According to this equation the two fillers concur to create the conductive network in an additive manner. For the hybrid composites under investigation we have calculated the first term of this equation for the volume fractions of fillers that are necessary to achieve the percolation threshold: namely 6.7 × 10^−4^ volume fraction (0.1 wt.%) of CNTs combined with 0.29 × 10^−2^ volume fraction (0.5 wt.%) of GNPs or 0.26 × 10^−2^ volume fraction (0.5 wt.%) of graphite, respectively. It was possible to verify that the first term of the equation at the percolation threshold of hybrid composites assumes values of about 0.5. In the case of the composites under investigation containing 0.05 wt.% of CNTs only, and containing the additional loads of 0.6 wt.% of GNPs or 1.5 wt.% of graphite, which are necessary to get to the percolation threshold, the first term of the equation became 0.3. Then in these composites the contribution given by the two fillers to the achievement of the percolation threshold was more than additive, which clearly demonstrates their synergetic interaction. This synergetic effect can be observed at very low total load of fillers, and using different CNT/second filler ratios.

### 3.4. Thermal Conductivity

**Figure 5:**
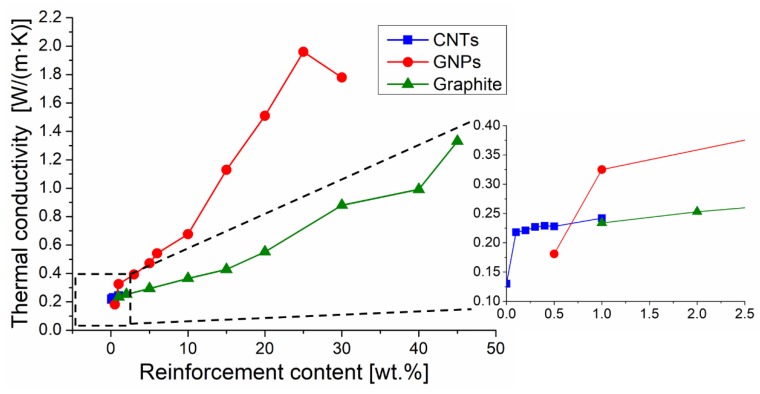
Effect of filler content on the thermal conductivity of composites.

The thermal conductivity of composites containing a single filler as well as that detected for hybrid composites are reported in Table 4. Figure 5 compares the effect of the increase of concentration of CNTs, GNPs, and graphite on thermal conductivity. 

The increase of CNT concentration from 0.1 wt.% to 1 wt.% resulted in a thermal conductivity enhancement of 11% only, while the same increment of CNT concentration caused an electrical conductivity increase of eight orders of magnitude. Therefore, CNTs had a very different influence on electrical and thermal conductivity. The limited effect on thermal conductivity with the addition of CNT up to 1 wt.% (0.67 vol.%) is consistent with literature data, that also show that thermal conductivity is poorly affected by the processing path. For instance, Gojny et al. [40] measured a thermal conductivity of 0.252 W/(m·K) on an epoxy-0.3 vol.% MWCNT composite processed by three roll mill while Yang et al. [30] processed by sonication an epoxy-1 wt.% MWCNT obtaining a conductivity value of 0.211 W/(m·K). It seems that the value of thermal conductivity of composites containing CNTs does not significantly depend on the processing method whether causing, or not, nanotube alignment. In fact, the capability of CNTs to improve thermal conductivity (which is potentially very high because of their intrinsic conductivity) is greatly limited by their tremendous surface area, and then by the strong phonon boundary scattering occurring at the interface with the matrix. On the other hand, the interfacial resistance can be lowered by properly functionalizing the CNTs [11].

Thermal conductivity appreciably increased, roughly in a linear manner, with the increase of concentration of both GNPs and graphite, but GNPs allowed for more quick conductivity improvement. For instance, the addition of 30 wt.% of filler to the epoxy resin resulted in a conductivity of 1.78 W/(m·K) and 0.88 W/(m·K) when GNPs or graphite were used respectively. Similar results should be observed when the same volume fraction of GNPs and graphite were added to epoxy matrix, since these two fillers have very similar densities. Then the conductivity improved with respect to the net epoxy matrix (thermal conductivity 0.13 W/(m·K) [28]) of about 1270% or 570%, respectively, when 30 wt.% of GNPs or graphite were used as fillers. This noticeable difference was expected on the base of the very different thermal conductivity of the GNPs (ranging between 400 and 5000 W/(m·K) [10,13]) and the graphite (298 W/(m·K) on the graphite planes but 2.2 W/(m·K) in the direction perpendicular to the graphitic planes [11]). However, the effect of GNPs depends also on other parameters: size and orientation of graphene plates, morphology of plates, and resistance of interfaces with the matrix. The positive effect of GNP alignment for thermal conductive applications has been reviewed by Zhang et al. [41]. Moreover the intrinsic thermal conductivity of GNPs is greatly affected by the presence of defects [41], and also depends on the platelet size. In fact, according to Raza et al. [21] the conductivity of a silicon-based composite with 25 wt.% of GNPs decreases from 3 W/(m·K) to 1.18 W/(m·K) when using plates 15 µm and 5 µm wide, respectively. Shen et al. [42] extrapolated the thermal conductivity of embedded graphene platelets with different lengths and found that the conductivity decreases down to a few hundreds of W/(m·K) when the platelet size is less than 100 nm. On the other hand, it is necessary to consider that the size of GNPs can be reduced during the composite processing, in particular when using a high speed mixer, while the three roll mill is considered a gentler dispersion method [22]. The importance of the resistance of the interface with the matrix is demonstrated considering that the intrinsic conductivity of GNPs can be even 16 times greater with respect to that of graphite. In addition, the conductivity of composites containing GNPs was about double that of detected when adding graphite. In fact, the addition of the same weight fraction (or volume fraction) of GNPs or graphite results in a very different extent of the interfaces between the filler and the matrix. As the specific surface area of GNPs is much higher than that of graphite, the interfacial thermal resistance should also be much higher when using GNPs. Then the higher interfacial thermal resistance of GNPs should partially counterbalance their superior intrinsic thermal conductivity. 

The formation of a conductive network when the GNP concentration reaches a threshold has been hypothesized [13], but Figure 5 shows that the thermal conductivity progressively increases with the increase of GNP or graphite concentration, and the trend of these curves seems a bit different from the typical “S” curves characteristic for the percolation phenomena. 

Several models for the prediction of the thermal conductivity of polymer composites filled with particles or fibers have been proposed [16,43,44], but they do not seem well suited for describing the nano-composites behavior. The series model (rule of mixture), that currently applies to composites with continuous fibers, could be tentatively used for composites containing aligned CNTs over the percolation threshold. However, this model greatly overestimates the composite conductivity because of the interfaces between interconnected nanotubes, and as not all the nanotubes concur to constitute the network. The parallel model as well as the similar Halpin-Tsai model could be adopted for predicting the conductivity of the composites containing nano-platelets. However, it is well known that the physical properties calculated according to these models only slightly differ from those of the matrix, in particular when the filler concentration is below 50%, while the addition of a very small quantity of GNPs appreciably increased the thermal conductivity. The Lewis and Nielsen semi-theoretical model includes the effects of the aspect ratio and the orientation of short fibers, therefore it seems tailored for composites containing CNTs.

The thermal conductivity could be calculated according to the following Equation (2):

(2) $$\lambda_{c}=\lambda_{m}\;\left[\frac{1+A\;B\;V_{f}}{1-B\;V_{f}\;\frac{\lambda_{f}}{\lambda_{m}}-1}\right];\;A=\frac{2\;L}{D};\;B=\;\frac{\frac{\lambda_{f}}{\lambda_{m}}-1}{\frac{\lambda_{f}}{\lambda_{m}}-A};\;\psi =1+\;\left(\frac{1-\Phi_{m}}{\Phi_{m}^{2}}\right)\;V_{f}$$

where *λ_c_*, *λ_f_*, and *λ_m_* are the thermal conductivities of the composite, the filler, and the matrix; *V_f_* is the volume fraction of filler; *L* and *D* are respectively the length and the diameter of the CNTs; $\Phi_{m}$ is a parameter taken equal to 0.82 for aligned fibers packaged in a random manner.

Assuming a thermal conductivity for MWCNT of 1000 W/(m·K) (significantly lower than the theoretical one because of the effect of the interfaces with the matrix and the possible presence of defects) the calculated thermal conductivity ranges between 0.157 W/(m·K) and 0.396 W/(m·K) for composites with a CNT concentration ranging between 0.1 wt.% and 1 wt.%. These calculated values were similar to the experimental ones (same order of magnitude), but according to this model the calculated thermal conductivity should increase with the CNT concentration more quickly than the experimental one did. However, it must be considered that the increase of porosity with the filler load should affect negatively the thermal conductivity.

On the contrary, the Lewis and Nielsen model is not at all suitable for predicting the thermal conductivity of composites with GNPs, regardless of the thermal conductivity of GNPs used for the calculation (one thousand or few hundreds of W/(m·K)). Anyway, this is not surprising because the platelets cannot affect the conductivity in the same manner of fibers or rods. 

**Figure 6:**
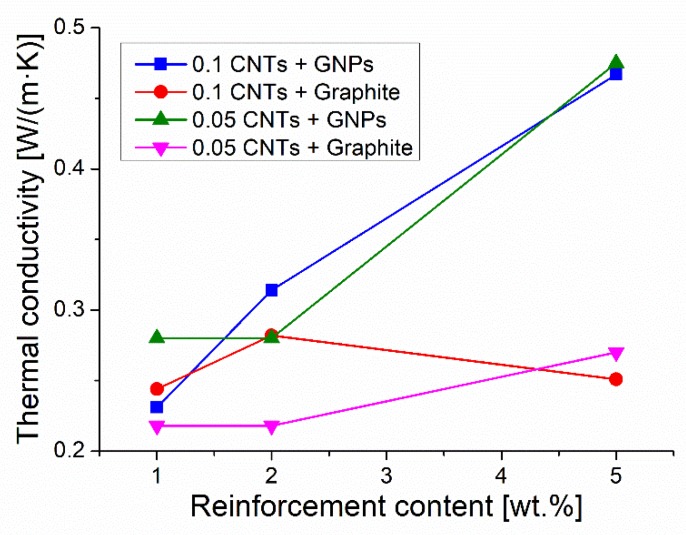
Effect of filler combinations on the thermal conductivity of hybrid composites.

In this paper, the possible synergetic effect of fillers with different aspect ratios on the thermal conductivity was also investigated by adding an increasing amount of GNPs or graphite to composites containing 0.1 wt.% or 0.05 wt.% of CNTs dispersed in the epoxy matrix. The results are depicted in Figure 6. 

When graphite was added to a composite containing 0.1 wt.% or 0.05 wt.% of MWCNTs the thermal conductivity did not increase, but these hybrid composites showed a conductivity even lower than the composites containing the same concentration of graphite alone. On the contrary, the combination of CNTs and GNPs resulted in a thermal conductivity enhanced with respect to that of the composites containing the same concentration of MWCNTs (0.1 wt.% or 0.05 wt.%). However, the adoption of GNPs alone as a filler (from 1 wt.% to 5 wt.%) granted better or similar conductivity with respect to the hybrid composites containing the same concentration of GNP and very small quantities of MWCNT (0.1 wt.% or 0.05 wt.%). Conclusively, hybrid composites did not show any thermal conductivity improvement with respect to composites containing one filler only (namely GNPs or graphite). Neither synergetic nor additive effect between the two fillers was observed in the present research. Nevertheless, the three roll mill technique allowed for the production of hybrid composites with better conductivity with respect to similar materials processed by sonication. For instance, hybrid composites with 0.1 wt.% CNTs + 1 wt.% GNPs, or 0.1 wt.% CNTs + 5 wt.% GNPs showed thermal conductivities of 0.23 W/(m·K) and 0.47 W/(m·K), respectively, while the literature reports for composites processed by sonication showed a conductivity of 0.19 W/(m·K) with 0.1 wt.% CNTs + 0.9 wt.% GNPs [19] and of 1.5 W/(m·K) with 5 vol.% CNTs + 5 vol.% GNPs [17].

Contrasting data about synergetic effect of nanofillers on thermal conductivity have been reported in the literature. According to some authors a synergetic effect between CNTs and GNPs is observed only when the total concentration of fillers exceeds 10 vol.% [17] or, for lower filler concentration (e.g., 1 wt.%), when functionalized CNTs are used [19]. On the contrary, according to Paszkiewicz et al. [35], no synergetic effect occurs in a wide range of total concentrations of these fillers (from 3 wt.% to 20 wt.%).

Probably the positive effect on thermal conductivity provided by a continuous network between fillers showing different aspect ratios can be overbalanced by the detrimental phenomena arising from the strong resistance of the interfaces filler/matrix and CNTs/GNPs. Moreover, the composite porosity very likely resulting from the difficulty of processing filler/matrix mixtures showing high viscosity, greatly hinders thermal conductivity of polymer-based composites.

## 4. Conclusions

The calendaring technique allowed for the production of composites with carbon-based filler homogeneously dispersed in an epoxy matrix. The conductivity of these composites was enhanced by optimizing the processing parameters. 

Noticeable electrical conductivity improvement was achieved owing to a conductive network formed as soon as the filler load reached the percolation threshold. The mechanism for thermal conductivity was not mainly affected by the formation of percolation paths instead.

Carbon nanotubes were more effective than graphene nanoplatelets for enhancing the electrical conductivity, while graphene nanoplatelets performed better than carbon nanotubes for improving thermal conductivity.

Hybrid composites containing very low concentrations of two fillers showing different aspect ratios (mainly CNTs and GNPs) displayed electrical conductivity six orders of magnitude higher than the net matrix. This behavior was attributed to the preferred orientation of the fillers, caused by calendaring, which resulted in a synergetic effect able to lower the percolation threshold.

Such a kind of synergetic effect was not observed for the thermal conductivity, since in this case no advantage arose from the combination of different fillers. The thermal resistance of interfaces between the filler and the matrix and between the filler particles, as well as the size of filler particles, dominate the heat flux.

## Figures and Tables

**Table 1:** Three roll mill trials for epoxy graphene-like nanoplatelets (GNPs) (2 wt.%) processing.

Processing Method (Trial)	Processing Parameters					Thermal Conductivity (W/(m·K) ± SD)
	Number of Steps	Gap 1 (µm)	Gap 2 (µm)	Contact Mode	Rpm (maximum)	
A	9	45	15	No	600	0.349 ± 0.021
B	3	45	15	No	600	0.375 ± 0.006
	3	15	5	No	600	
C	9	45	15	No	600	0.338 ± 0.014
	9	15	5	No	600	
D	3	45	15	No	600	0.365 ± 0.018
	3	15	5	No	600	
	3	0	0	Yes	300	
E	9	45	15	No	600	0.352 ± 0.028
	9	15	5	No	600	
	3	0	0	Yes	300	
F	9	45	15	No	600	0.310 ± 0.034
	9	15	5	No	600	
	6	0	0	Yes	300	

**Table 2:** Density of composites with different filler content.

Filler	Filler Concentration (wt.%)	Experimental Density (g/cm^3^)	Experimental versus Theoretical Density (Ratio)
No one (net epoxy)	-	1.1496 ± 0.0001	>0.999
MWCNT	0.1	1.1496 ± 0.0001	>0.999
MWCNT	0.5	1.1041 ± 0.0001	0.959
GNP	1.0	1.1092 ± 0.0003	0.960
GNP	10.0	1.1482 ± 0.0001	0.956
GNP	25.0	1.1450 ± 0.0007	0.890
Graphite	20.0	1.0499 ± 0.0005	0.896
Graphite	40.0	1.2026 ± 0.0009	0.846
MWCNT + GNP	0.1 + 2	1.0913 ± 0.0001	0.941
MWCNT + GNP	0.1 + 5	1.1754 ± 0.0004	0.910
MWCNT + Graphite	0.1 + 2	1.0919 ± 0.0007	0.940
MWCNT + Graphite	0.1 + 5	1.1129 ± 0.0002	0.944

**Table 3:** Electrical conductivity of the two-components and the hybrid (three components) epoxy-based composites.

Sample Composition (wt.%)				Electrical Conductivity S/m
MWCNT	GNP	Graphite	Epoxy	
0	-	-	100	<1 × 10^−9^
0.1	-	-	remainder	<1 × 10^−9^
0.2	-	-	remainder	5.18 × 10^−5^
0.3	-	-	remainder	5.97 × 10^−4^
0.4	-	-	remainder	5.08 × 10^−3^
0.5	-	-	remainder	1.20 × 10^−2^
1.0	-	-	remainder	1.21 × 10^−1^
-	From 0.5 to 5.0	-	remainder	<1 × 10^−9^
-	6.0	-	remainder	2.44 × 10^−6^
-	7.0	-	remainder	5.93 × 10^−6^
-	9.0	-	remainder	6.80 × 10^−6^
-	10.0	-	remainder	2.71 × 10^−6^
-	15.0	-	remainder	9.20 × 10^−5^
-	20.0	-	remainder	2.14 × 10^−4^
-	25.0	-	remainder	1.64 × 10^−1^
-	30.0	-	remainder	1.62 × 10^−1^
-	0	From 1.0 to 30.0	remainder	<1 × 10^−9^
-	0	40.0	remainder	2.02 × 10^−5^
-	0	45.0	remainder	1.92 × 10^−4^
0.1	1.0	-	remainder	1.20 × 10^−3^
0.1	2.0	-	remainder	1.32 × 10^−3^
0.1	5.0	-	remainder	5.43 × 10^−4^
0.1	-	1.0	remainder	3.57 × 10^−5^
0.1	-	2.0	remainder	1.65 × 10^−4^
0.1	-	5.0	remainder	1.61 × 10^−4^
0.05	1.0	-	remainder	6.02 × 10^−5^
0.05	2.0	-	remainder	1.25 × 10^−4^
0.05	5.0	-	remainder	3.29 × 10^−4^
0.05	-	1.0	remainder	<1 × 10^−9^
0.05	-	2.0	remainder	3.99 × 10^−5^
0.05	-	5.0	remainder	7.37 × 10^−5^

**Table 4:** Thermal conductivity of the two-components and the hybrid (three-components) epoxy-based composites.

Sample Composition (wt.%)				Thermal Conductivity (W/(m·K) ± SD)
MWCNT	GNP	Graphite	Epoxy	
0	-	-	100	0.130 ± 0.004
0.1	-	-	remainder	0.218 ± 0.002
0.2	-	-	remainder	0.221 ± 0.002
0.3	-	-	remainder	0.227 ± 0.001
0.4	-	-	remainder	0.229 ± 0.001
0.5	-	-	remainder	0.228 ± 0.001
1.0	-	-	remainder	0.242 ± 0.001
-	0.5	-	remainder	0.181 ± 0.003
-	1.0	-	remainder	0.325 ± 0.004
-	3.0	-	remainder	0392 ± 0.006
-	5.0	-	remainder	0.472 ± 0.004
-	6.0	-	remainder	0.541 ± 0.004
-	10.0	-	remainder	0.676 ± 0.007
-	15.0	-	remainder	1.129 ± 0.010
-	20.0	-	remainder	1.511 ± 0.017
-	25.0	-	remainder	1.960 ± 0.022
-	30.0	-	remainder	1.784 ± 0.027
-	-	1.0	remainder	0.234 ± 0.002
-	-	2.0	remainder	0.253 ± 0.001
-	-	5.0	remainder	0.294 ± 0.002
-	-	10.0	remainder	0.365 ± 0.003
-	-	15.0	remainder	0.428 ± 0.001
-	-	20.0	remainder	0.552 ± 0.002
-	-	30.0	remainder	0.880 ± 0.008
-	-	40.0	remainder	0.992 ± 0.015
-	-	45.0	remainder	1.331 ± 0.001
0.1	1.0	-	remainder	0.231 ± 0.006
0.1	2.0	-	remainder	0.314 ± 0.004
0.1	5.0	-	remainder	0.467 ± 0.004
0.1	-	1.0	remainder	0.244 ± 0.002
0.1	-	2.0	remainder	0.282 ± 0.002
0.1	-	5.0	remainder	0.251 ± 0.001
0.05	1.0	-	remainder	0.280 ± 0.004
0.05	2.0	-	remainder	0.280 ± 0.004
0.05	5.0	-	remainder	0.475 ± 0.005
0.05	-	1.0	remainder	0.218 ± 0.002
0.05	-	2.0	remainder	0.218 ± 0.002
0.05	-	5.0	remainder	0.270 ± 0.001
